# Exploration of Multilevel Barriers and Strategies That Affected Early COVID-19 Vaccination and Testing in Rural Latino Communities in Southwest Florida

**DOI:** 10.3390/ijerph191811785

**Published:** 2022-09-18

**Authors:** Acadia W. Buro, Kevin Roman Candelaria, Rocio Bailey, Frances Luna, Alexandra Albizu-Jacob, Marilyn Stern, Laura Redwine

**Affiliations:** 1 Department of Health Outcomes and Behavior, Moffitt Cancer Center, Tampa, FL 33617, USA; 2 Department of Child and Family Studies, College of Behavioral and Community Sciences, University of South Florida, Tampa, FL 33612, USA; 3 Hispanic Services Council, Tampa, FL 33614, USA; 4 Department of Family Medicine and Community Health, Miller School of Medicine, University of Miami, Miami, FL 33136, USA

**Keywords:** COVID-19, Latino, rural, pandemic response

## Abstract

The COVID-19 pandemic has disproportionately impacted multiple racial and ethnic minority groups, including Latinos residing in rural communities. Low rates of vaccination and testing combined with social determinants of health have contributed significantly to this disparate impact. Given the needs and constraints unique to rural Latino migrant and immigrant communities, this qualitative study examined multilevel barriers and strategies that affect COVID-19 vaccination and testing uptake among these communities in southwest Florida. Four focus groups (n = 25) were conducted between March and April 2021 with various key stakeholders, including rural Latino community members, local leaders, and community health workers (‘Promotoras de Salud’). Themes that aligned with barriers to COVID-19 vaccination and testing included fear, lack of control, misinformation, lack of accessibility, and institutional/policy issues; themes that aligned with strategies to improve COVID-19 vaccination and testing uptake included faith, taking care of self, and community and family resilience. Recommendations for improving future pandemic responses for rural Latino communities include incorporating multiple levels of intervention, such as consideration of the role of the family, involving trusted community members, and ensuring the development and implementation of fair and consistent policies.

## 1. Introduction

Historically disadvantaged populations, including Latino communities [1] and migrant workers [2], have been disproportionately affected by the COVID-19 pandemic. Social determinants that put Latino populations at an increased risk of COVID-19 include co-occurring medical conditions, barriers to healthcare access, immigration status, language barriers, and type of employment [3]. Although the US Latino population is a diverse ethnic group consisting of South or Central American, Puerto Rican, Cuban, Mexican, or other Spanish culture, Latinos in general are more likely to work in frontline situations, including essential jobs in farming, food production, grocery stores, waste management, cleaning and sanitation, hospitality, and other high-transmission-risk industries [4]. Low-socioeconomic individuals employed in such jobs, which often require on-site attendance, limited access to personal protective equipment, and prolonged close contact with others, accounted for the majority of COVID-19 deaths in the first year of the pandemic [5]. Moreover, structural, institutional, and individual discrimination (e.g., policies, racism) can increase exposure to social determinants that put Latino populations at high risk of COVID-19 and poor long-term health consequences, including obesity, diabetes, hypertension, and heart failure [6]. Additional socioeconomic factors contributing to crowded living conditions, poor nutrition, and less access to medical care may further contribute to COVID-19 risk among rural Latino populations [7].

Vaccination and diagnostic testing are critical to controlling outbreaks, yet rural Latino populations have greater rates of COVID-19 vaccine hesitancy [8,9] and barriers to testing compared to non-Latino whites [10]. According to the Centers for Disease Control and Prevention (CDC), data collected through May of 2022 indicate that compared with non-Latino whites, Latinos have 1.5 times the number of COVID-19 cases, over two times the number of hospitalizations, and 1.8 times the number of deaths [11]. Various predictors of COVID-19 vaccine uptake in the general population include vaccine knowledge, household income, perceived risk and severity, confidence in the scientific community, and acceptance or rejection of conspiracies associated with COVID-19 vaccinations [12,13]. Moreover, a history of mistreatment by the healthcare system in Latino communities has led to an associated distrust, influencing COVID-19 vaccine uptake [8,14]. Several barriers to testing for Latino communities residing near the United States-Mexico border have been identified, including fear and myths related to the medical system and the government [10]. As experiences may vary by region due to differences in state-wide COVID-19 policy and local actions, there is a need for research investigating multi-level determinants of COVID-19 disparities in rural Latino populations in various regions of the US to inform future pandemic and disaster response.

In Florida, 26% of the population identifies as Latino [15], and Latino populations were most likely to have contracted COVID-19 at the time this study was conducted [16]. Florida first received doses of vaccines for COVID-19 in December 2020, and distribution to the general population began in February 2021; individuals aged 60 and older were deemed eligible to receive the vaccine on 15 March 2021, and all Florida residents aged 18 and older were considered eligible on 5 April 2021 [17]. Proof of Florida residency was initially required for COVID-19 vaccination; the order was rescinded on 29 April 2021 [18]. However, rural regions experienced ongoing structural and access barriers, contributing to low vaccine uptake among Latinos [19]. Therefore, feedback from community members and other key stakeholders is critical to inform public health practice and policy.

This study aimed to examine the barriers and strategies affecting COVID-19 testing and vaccine uptake at the individual, interpersonal, institutional (e.g., healthcare system), and community levels in southwest Florida rural Latino migrant and immigrant communities during the height of COVID-19 lockdown and the rollout of the COVID-19 vaccine. Information gathered from the present study can inform policy for vaccination and testing as the present pandemic continues, and for potential future pandemics.

## 2. Materials and Methods

### 2.1. Research Questions, Study Design, Setting, and Participants

**Figure 1:**
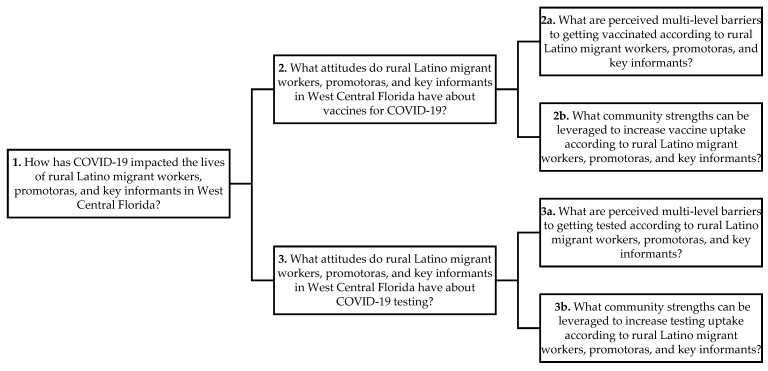
Research questions to examine the barriers that affect COVID-19 testing and vaccine uptake at the individual, interpersonal, institutional (e.g., healthcare system), and community levels in southwest Florida rural Latino migrant and immigrant communities.

To address the study research questions on the impact of COVID-19 on the lives of rural Latino migrant workers, Promotoras de Salud (referred to as promotoras, i.e., community health workers) and key informants in southwest Florida were enrolled (Figure 1). We performed a qualitative study that included focus groups conducted between March and April 2021, each lasting approximately two hours. Focus groups were chosen as the methodology to allow facilitators to encourage honest discussion in a safe setting among participants who have had shared experiences. Our target was four groups of approximately six to eight participants per group to achieve saturation [20]. Three focus groups (community members and promotoras) were conducted in person and in Spanish. One (key informants, i.e., local leaders) was conducted via Zoom in English. The focus groups were audio-recorded and transcribed verbatim, and Spanish transcripts were translated into English. Informed consent was obtained from all participants according to University of South Florida Institutional Review Board (IRB) specifications.

Latino community members, key informants, and promotoras from rural southwest Florida (n = 25) were recruited through Hispanic Services Council (HSC), a community-based organization offering resources and advocacy for Latinos. HSC hires promotoras, community health workers who serve as the bridge between their community and culturally appropriate outreach services and health education, to serve their community [21]. Participants were selected by the HSC project director and promorota program manager because they self-identified as stakeholders who were representative of the broader community as rural Latino residents with similar backgrounds to the surrounding community who could benefit from improvements to the community based on the findings of this and other studies relating to COVID-19. Specifically, participants were recruited through flyers on the HSC website and at HSC events, word of mouth, and direct contact by HSC staff based on pre-existing knowledge of community leaders and other key stakeholders.

Focus groups with community members (n = 6 and n = 7) and promotoras (n = 7) were facilitated by the HSC promotora program manager (F.L.). The focus group with key informants (n = 5; local leaders involved in migrant services, education, and faith communities) was facilitated by the HSC project director (R.B.). Participant inclusion criteria included: (1) men and women identifying as Latino; (2) aged 18 or older; (3) living in one of the four target communities (Dover, Plant City, Ruskin, and Wimauma); and (4) ability to understand and provide consent for participation in the study. The only exclusion criterion was the inability to speak English or Spanish, depending on the group.

### 2.2. Analysis

Audio from the focus group meetings were recorded and transcribed verbatim. Although confidentiality cannot be guaranteed in the group setting, provisions were made to protect anonymity and confidentiality, ensured by omitting names, emails, telephone numbers, and mailing addresses from the data analysis and by analyzing and reporting data in aggregate. Focus groups were conducted in a private setting or HIPAA compliant Zoom meetings. Transcripts and notes were stored in a shared folder accessible only to our study staff.

After becoming familiarized with the data, A.W.B. and K.R.C. created a codebook. Three a priori codes were identified from interview questions, and sub-codes emerged through reading and coding transcripts and discussing coding decisions. Data were segmented using response as the unit of analysis. Each transcript was double coded by A.W.B. and K.R.C. in Dedoose software version 9.0 (Los Angeles, CA, USA). After coding each transcript, the coders calculated interrater reliability in Dedoose and discussed and resolved any discrepancies by clarifying codebook definitions. Cohen’s kappa was 0.9 by the fourth transcript (ranging from 0.7 to 0.9), indicating excellent interrater agreement. Segmented data were extracted to matrices in Microsoft Excel for each research question, and thematic analysis was applied to identify common themes in responses. A combination of an inductive and deductive approach was used. The codebook was informed by the social-ecological model [22] to examine multi-level barriers to COVID-19 vaccine and testing uptake (i.e., individual, interpersonal, institutional, and community/policy). Focus group transcripts were reviewed, discussed, and coded in an iterative process until agreement was reached on cross-cutting themes from the data that aligned with the research questions.

## 3. Results

### 3.1. Barriers

Eight overarching and cross-cutting themes emerged that speak to the barriers and strategies affecting COVID-19 testing and vaccination uptake. Five barrier-related themes were identified, including fear, lack of control, misinformation, lack of accessibility, and institutional/policy issues. Three themes related to strategies for improving COVID-19 testing and vaccination rates included faith, taking care of self, and community and family resilience.

Fear: Fear emerged as a salient theme among participants when asked about potentially contracting COVID-19. For example, participants mentioned fear of dying alone in the hospital, without loved ones allowed to visit, as illustrated by the following quotes:

*People believe, as [she] says, that when they go to a hospital, when they see the doctor, they’re too afraid. They’ve called me and said, “I’m very ill. I’m breathing badly”. I’ve said, “Please go see a doctor. This is an emergency. Call 911”. They say, “No, no, because if I go to the hospital, I’m going to die, and my family won’t even be able to say goodbye to me”.*

—Promotora

*That’s the number one concern that they’re not going to have anyone there. That they’re going to die alone and that the only thing their families are going to be given are their remains. People have that fear. That’s their fear when they get COVID. These are the concerns that I’ve seen and that they’ve called me about. Because unfortunately, every day, more and more people die of COVID. It’s dramatic not to say goodbye to your family. That’s the worst thing that can happen.*

—Promotora

In all four focus groups, fear appeared as an individual-level barrier to vaccination and testing, including fear of the vaccine and its side effects. For example, community members discussed how their fear contributed to mistrust, as illustrated in the following quote:

*Because there are so many rumors, so many things that have been said. Lately, some people were saying that they were giving them water, that they weren’t giving them anything. All that caused a little bit of fear. Another thing is, sometimes, you don’t know. Since they’re just coming out, we don’t know exactly what the consequences of the vaccine will be. Well, right now, they came out with a new dose. Everything is news and news, and honestly, you never know what’s true. They took it off the market because of what was happening, and that’s why sometimes, you have a little fear. When I went to get the vaccine, when I saw that the man already had the syringes ready. I didn’t see where he got it from, whether he really took it from the vaccine, or I don’t know what he put in me. Do you understand my fear? Because they’re not showing you, “Look, this is where I’m taking it from”.*

—Community member

Regarding testing, participants discussed the fear of getting a positive COVID-19 test result. For example, one promotora described how community members experience fear of stigma and inability to work related to a positive test result:

*Well, they wanted to go out and because they needed it at work. There were entire trailer communities with the virus. It was very sad because they didn’t know where to get it done. They were a little afraid and didn’t want to know they had the virus. They felt that rejection of, “If I say I have the virus, I’ll be rejected”. They didn’t want to say it.*

—Promotora

Community members also discussed fear of presenting documentation as a barrier to getting vaccinated or tested:

*So, I think, in our population, it’s more the fear of having to present documentation to get their vaccination. So, am I going to be asked to have an ID, or a birth certificate, or something like that? So, that’s the same reason they’re—they don’t come to schools and want to enroll their children is because they don’t have that documentation.*

—Community member

*They’re afraid immigration will find out. That’s why they don’t even want to register the kids in school because they’re fearful that immigration will get the information. They don’t want to register their information for the vaccinations. That’s what (name redacted) said at the very beginning.*

—Key Informant

Lack of control: Lack of control was an emergent theme in all focus groups regarding the impact of COVID-19 on everyday life. Participants described negative situations regarding work and school that were outside of their control. For example, participants described the burden on parents, as described in the following quotes by a community member and key informant:

*I mean, they don’t have access to the technology, so you have to do that face to face, and you have to go and meet them in their home, so you have to help them. And if there was—we were being told we couldn’t go anywhere, yet we were asking the parents to have this technology available. So, it was very frustrating, and to this day, it still is very frustrating because we make a lot of assumptions that our parents are ready to handle all this, and they’re not. Through no fault of their own. It’s just it’s kind of like a vicious circle, but going back to the positive, it’s at least now we know what we didn’t know. So, how we handle it in the future is a game-changer, and we can’t just sit and cross our arms and do nothing. It’s everybody’s responsibility to fix it.*

—Community member

*If a child is sick or showing any symptoms of anything, they go down to the clinic; parents have to pick them up. They cannot come back to school until they’ve had this COVID test, right? So, you’re hoping for a fast COVID test that the parent has access to, but not all the parents have access to these fast COVID tests because the idea is if the child comes up positive, then you’ve got to quarantine every child that that child’s been in contact with, which has been—had a huge impact this school year on the days of school that children have missed all across the whole school, and teachers, and everything.*

—Key informant

Participants also described the impact on work, including having to continue to work, sometimes without payment, or losing their job altogether, as illustrated by the following quotes:

*Restaurants were closed. Bars were closed. Whereas essential work, such as farm work, which I also do, is essential. You couldn’t stop working. You had to continue. Construction work, which I also do, also couldn’t be stopped. There was social distancing and everything, but you had to continue. Those types of jobs were essential. Government work, or work in the state health department, wasn’t considered essential. Unfortunately, those people didn’t have any form of income, so it affected them very hard.*

—Key informant

*At work, I never saw a case like that. In the case of my daughter, who works at Amazon, they told her that, since she tested positive, she had to stay home for a month. They only paid her for one week. The other weeks, she didn’t get paid.*

—Community member

*I worked with a family, and the wife was giving me their details, and the husband had a horrible cough. People also told me, “I was cooped up in my home for a month and a half. I have no job”. Someone else told me, “Look, I don’t have the same phone number because I was very sick for three months. I’m better now, but I don’t have anything. I had to start again from scratch.”*

—Promotora

*So, when you’re living day to day and every single paycheck is what you need in order to survive, taking a day off to get your vaccine, and then taking another day off to get your vaccine, and then thinking, “Maybe I’ll get sick, I might need another day off to get the vaccine”, is complicated. It’s not something that they can take lightly. And so, if the vaccines were offered closer to their homes because maybe they don’t even have transportation and they have to get to the source of the vaccine.*

—Key informant

Misinformation: Participants discussed misinformation and myths regarding the vaccines and testing. For example, as one promotora said, “What I’ve heard is that the (test) results aren’t accurate”. Promotoras and community members also discussed myths about vaccines and exterminating or sterilizing certain populations, as illustrated by the following quotes:

*That’s why they’re giving (vaccines) first to 65-year-olds. That’s what they say. Because they want to exterminate old people, so that they will no longer be in the way. What did a lady tell me? Oh yes, that it’s so that they will no longer have to pay their Social Security or their pensions. That they need to renew the race.*

—Promotora

*The other thing is that everybody will be sterilized because, of course, they don’t want certain populations to keep growing. Matter of fact—and other people they just—they just die, and that’s not being publicized. So, there is quite a bit of bad information out in the different communities, especially the ones that are more isolated than others.*

—Community member

Some participants mentioned how misinformation led to fear. As one community member described:

*But yes, what I see mostly, it’s because of fear. When we call them, they are scared. Even the last time we had a gentleman that went in, he was very ill. He didn’t want to get tested because he said that his little boy told him that if he gets tested, they were going to put a chip on his nose. So, I guess it’s miscommunication, the education, and the fear.*

—Community member

Participants also discussed how misinformation spreads in their communities, including through social media and the news.

*Because they’re afraid due to misinformation, I think. They have to be educated on it to get rid of all those myths that they have and all the beliefs. As [she] says, technology is overtaking us, so everything you see on Facebook, everything you see is that they’re going to steal our DNA, and this, and that.*

—Promotora

Lack of accessibility: Lack of accessibility was a salient theme regarding COVID-19 vaccines and tests. Community members described a lack of time and resources as barriers, as illustrated by the following quotes:

*Access, access, access because whether it is exactly what you say, and yes, I have family, and I know how much they struggle to get their own, and that’s why every single time I’m like, “And we expect our farmworkers to go online and have on their little phone, and they put in a program or something so that you can eventually get it”. It is very unrealistic, so the access to the vaccines was a total failure in Florida, period.*

—Community member

*But for our families, to expect them to be able to access, to register, and get in, is very complicated for them. And to set up those appointments. It’s not simple. And it’s time-consuming. And it’s just what they don’t have. They need a more ease of access. So, that’s one more thing that has prevented them from getting their vaccination.*

—Community member

Some participants described how difficult it was for them to get a test. As one community member explained:

*…It was not easy to find a place and find an appointment. We wanted the rapid test because I wanted to be able to come back to work. It was right around Christmas time, and to find the rapid test was—I mean, I had to get up at like three in the morning and go stand in line or wait in my car and just pray to God that I got a test, and I didn’t want to wait for three days, so I was very—I and my entire family, we were very frustrated because I’m very tech-savvy. I can find anything on the internet, but it was not easy for me. So, it’s hard. It’s not easy.*

—Community member

Lack of transportation was another emergent sub-theme regarding accessibility:

*They don’t have transportation, so I think that would be good. For the country or the state to do something like have healthcare workers go from house to house, knocking on doors.*

—Promotora

Institutional/policy issues: Various institutional and policy issues were discussed as barriers to vaccination and testing. For example, participants described how institutions requiring documentation were a barrier to vaccination:

*Only people over 65 who are residents of Florida have been able to get it. For those who are here, for example, and who stayed because they couldn’t travel due to COVID, they don’t have an ID, or they’re not Florida residents, or they don’t have a phone bill, electric bill, anything. They lack proof that they’re Florida residents. They haven’t been able to get it. Immigrants haven’t been able to get it because I’ve called. Those who don’t have papers.*

—Promotora

*It’s very hard for them to do anything, and they do not. Their employers are not willing to give them check stubs. They’re here undocumented. They don’t have social security numbers. They’re working under the table. So, most of our Mexican families are moving along, but this huge population that’s coming in, and I mean it’s big, and it’s growing. It is really a place where we need to provide a lot of support.*

—Community member

Another community member mentioned the presence of law enforcement at testing sites as a barrier:

*And national guard soldiers. They’re not holding guns or anything like that, but they’re—it’s a big presence. So, they’re not—so, when you do these community events, I understand you have to have law enforcement, but do they have to be in full-fledged uniform because my families will shy away from that. I’m hoping that changes with the changes with what’s kind of changing right now. We’re going—I don’t want to get political, but that’s where I’m going.*

—Community member

The high cost of tests was discussed as another institutional barrier. For example:

*What’s the business, then? The fact that the free places where they’re being done make you go somewhere else, where they charge you 150 dollars. Then, it turns out that your test is negative. That’s a huge point because here, everything is a business, in my opinion”.*

—Promotora

Another community member discussed the lack of distribution of resources for migrants:

*Whatever or whoever they are, and I met with every person that I could with the CEOs, and the this, and the that, and they all had their good excuses. And all across the state, I’m not going to say just here, I’m saying all across the state was the same resources, resources, resources. And I’m like, “But hey, you’re getting federal dollars for migrants”, and but they still were never on their list.*

—Community member

Participants also reflected on institutional and policy issues when discussing the general impact of COVID-19. As one key informant stated, “Yes, in terms of the work they do, they are (essential). Now, when it comes to the vaccine, they aren’t.”

### 3.2. Strategies

Faith and hope for the future: Faith and hope for the future was an emergent theme regarding the vaccine, which was discussed by key informants and promotoras as illustrated by the following quotes:

*Injection, and for many, I think it’s a light at the end of the tunnel, right? To hear about a vaccine for this disease, it’s a light at the end of the tunnel for many. Also, for many, I think that the community is among those who fear side effects from the vaccine and everything. There’s rumors, and a lot of people don’t want to be vaccinated, but in terms of the vaccine itself, it’s a light at the end of the tunnel.*

—Key informant

*There’s a lot of pain, but we’re here, and we have hope with the vaccine. We say that even if we’re vaccinated, we’ll still wear our masks and everything. I hope we’ll be a little calmer now, though, because we believe that now, we’re a little more protected.*

—Promotora

Participants described their faith in a higher power and prayer when discussing helping others with COVID-19 and the overall impact of COVID-19 on their lives. As one community mentioned, “I trust God. He’s the only one. Because I don’t trust the others”. One promotora discussed prayer as a way to connect with community members:

*It’s very hard to hear all that because we’re used to working with our people. We feel for them and their situations, and it’s very hard for us to give them what we used to give them. Sometimes, that’s so important. She and I used to work together a lot, and sometimes, people came to us crying, and we would hug them. Now, we can’t have that contact where they’re crying, and we hug them and tell them that we’ll pray for them.*

—Promotora

Taking care of self: Despite events that were outside of their control, many participants discussed how the pandemic forced them to take care of themselves in different ways, from wearing masks and social distancing to finding innovative ways to make money and alternative treatments for protection from the virus.

*…There’s very little, let’s say, culture in terms of self-care. COVID has taught us through that vulnerability that infections, diseases, obesity, a lot of problems that humanity faces, will never be addressed or treated. Now, with the emergence of COVID, people are worrying more so about themselves, taking care of themselves, protecting themselves. How many infections are around us? It’s not the first time that it exists. There have been a lot of types of diseases, even when H1N1 came out, there was a controversy that was maybe the closest thing we’ve had to face.*

—Promotora

*I can’t tell you that I saw them doing anything. A lot of people that I saw in the community made food to sell in the office or on street corners. Maybe to take it to the farm workers. They cleaned houses. Anything. Garage sales. No one wanted to be close to anyone else because of hygiene, but they created their own source of work. Whatever they could do.*

—Key informant

*Older people who decided not to go to the hospital and spent time at home looking for herbs. I’m telling you, they ran out of all the herbs that people use. You couldn’t find them in any store. People were practically trafficking those little herbs and ginger. All that had run out. There wasn’t any in any store.*

—Promotora

Community and family resilience: Participants described how families and communities had worked together differently, exemplifying resilience during a crisis. Community members discussed looking out for each other. For example, community agencies have come together, and specific community locations have served as “safe places” for those who do not have documentation:

*COVID has caused for a lot of different agencies and communities to come together and really start figuring out or start voicing our concerns and really focusing on what are our rights as—not even as citizens, as human beings.*

—Community member

*Families went, and they didn’t have the ID; they were not tested. And this is when they were in some of those locations that were safe places for them to go, so which is why, and, again, I do plan to go and—because I want to know what it is they’re requiring or not.*

—Community member

Community members also described how the pandemic highlighted inequities and gave a voice to marginalized communities. Community members discussed that, although they have always supported each other, the pandemic provided an opportunity for decision makers to hear marginalized populations’ experiences, as illustrated by the following quotes:

*COVID has a lot of negative impacts, but it also has—it’s like the slingshot that has pushed us in the right direction. So, I love hearing all this conversation because we all represent different communities or different agencies, and I’m—I think we’re going to start seeing some changes, and people really noticing that, yes, our essential workers, even the most marginalized of populations, we really get—they got tossed aside.*

—Community member

*In my eyes, COVID has put a very strong light on the inequities that our Latinos and especially our migrant population is going—so, it’s not—to me, it’s a positive because I am getting more traction with even within our school system. I’m getting more traction on issues that have plagued our families for forever. I’ve been an educator for [decades] all here in Hillsborough County, and I’ve always been with the migrant—supporting the migrant population in one way or form, but—so, COVID has really provided a venue for our voices to be heard.*

—Community member

Community leaders and promotoras discussed ways in which they took the initiative to increase vaccine access in rural communities, as illustrated in the following quotes:

*We have taken on finding vaccinations for our families because that has been a nightmare in this state. I’m sure everybody knows that. But we have been able to, a trickle at a time, get some of those vaccines out into the rural communities and some out into the fields. And, as of today, hopefully, with this new change that, they will not be requiring certain information. We are really hoping that more families can be vaccinated. If somebody chooses not to do it, that’s okay, but we just want to make sure that the opportunity is there, and so far, most of our families have been wide open.*

—Community member

*I, personally, have helped several senior citizens make appointments because they don’t know how to really speak up for themselves. I’ve helped them, and some of them, I haven’t been able to get an appointment for. I register them. I call a line. I register them. I give them my phone number. They call me, and then they’re registered. Well, they call me and ask me for all the information.*

—Promotora

Family strength and resilience were highlighted as well. Some participants mentioned that spending more time together as a family was a silver lining of the pandemic.

*It’s been very rough. No one expected this. Everything completely collapsed in a very bad way. At the same time, there were also beautiful things. More family time. Being closer to my family and children. Maybe before, we’d leave for work, and we didn’t see each other. We didn’t sit down at the table or anything. Now, with COVID, we became closer as a family.*

—Key informant

*I see the positive side of COVID. It’s brought a lot of bad things, but it’s also generated positive things. Family time let’s say. We also have an approach to information technology, and that has allowed us to spend more time with our family. We’ve also started working from home because a lot of mothers need to work from home to take care of their children. Fathers, too. We have to see the positive side as well, I think.*

—Promotora

Others described doing what they had to do to take care of their family, as exemplified by the following quote from a key informant:

*I had two jobs. I worked in a church. I worked at a restaurant, and on Wednesdays, I worked in a church. Then, I lost that job during the pandemic. Now, I don’t know when they’re going to open again. I said, “Wow, how am I going to make it?” I have three children. My husband doesn’t work. I said, “No.” I started doing what I had to do.*

—Key informant

## 4. Discussion

This paper qualitatively explored attitudes about COVID-19 vaccines and testing from rural Latino migrant community members, promotoras, and key informants in southwest Florida, including perceived multi-level barriers and strategies to increase vaccine and testing uptake during the height of COVID-19 lockdown. Themes aligning with barriers to COVID-19 vaccination and testing included fear, lack of control, misinformation, lack of accessibility, and institutional/policy issues; themes aligning with strategies to improve COVID-19 vaccination and testing uptake included hope for the future and faith, taking care of self and community, and family resilience.

Our findings of individual and interpersonal barriers, including fear, mistrust, and misinformation, are consistent with other investigations of rural Latino communities that reported generalized worry regarding COVID-19 but also a wariness of information from commercial news sources about COVID-19 [23]. For example, a study in Texas also found that fear, mistrust, and misinformation were barriers to testing in underserved Latino communities [10]. These findings suggest that rural Latino communities in diverse states experienced similar barriers to vaccination and testing during the pandemic and that the confusion that ensued during the beginning of the vaccine rollout due to misinformation likely influenced vaccine and testing hesitancy and indicate a need to leverage reliable, trusted sources of information in rural communities during pandemic and disaster responses. Mistrust and misinformation have also been identified as key barriers to COVID-19 vaccination in the general population [24].

Various community and institutional barriers were also identified in our study, including a lack of available and accessible appointments and identification requirements. Documentation status was also identified as a barrier to COVID-19 federal relief programs, such as stimulus checks and rental subsidies, among rural Latino communities, which could negatively affect living conditions (e.g., crowded living situations and food insecurity) associated with job loss during the pandemic [25]. Key informants in our study described how farmworkers are not treated as essential, despite their work being classified as “essential work,” putting them at higher risk of infection. This finding indicates a need for more consistent policies and supportive, safe work environments. Although individual- and interpersonal-level barriers such as fear and misinformation have been highlighted, prior research and news reports have also described harmful and unsafe working conditions among undocumented Latino migrant laborers despite their categorization as essential workers [25].

The most salient theme that reflected strategies to overcome these barriers was community and family resilience, followed by taking care of self and having hope and faith. Participants in our study discussed taking care of themselves and others by adhering to guidelines such as wearing masks and using alternative methods such as herbs. Previous research also found that using natural medicines to maintain health was a prevalent theme among rural Latino communities [23]. Community and family resilience were discussed in all four focus groups. This theme is consistent with prior literature suggesting that successful pandemic responses in vulnerable communities should build on existing trusted community organizations and community strengths [26]. In addition, our study highlighted that rural Latino communities in southwest Florida were empowered to take collective action and take care of each other (e.g., helping each other make vaccine appointments, highlighting community issues through the local school system). For example, participants in our study discussed community agencies working together as a strategy to increase vaccine and testing uptake. Community members described how the pandemic was an opportunity for their voices to be heard. These results highlight that, despite the numerous barriers identified in this study, rural Latino communities have strengths that should be leveraged in pandemic and disaster response, including community resilience to withstand inequities and crises and collective action to solve problems within the community.

Our findings suggest that family strengths should be considered in addition to community factors. Participants discussed aspects of family resilience, including the importance of taking care of one another and perceiving family time as a silver lining to the pandemic. As Latino children may be especially vulnerable to the physical and psychological effects of the pandemic due to structural inequities such as chronic poverty and a lack of culturally effective services, family union and resilience may be important considerations for future pandemic and crisis responses to link families to culturally appropriate resources that address food and housing security, immigration needs, and unemployment support [27]. Targeting families rather than individuals may increase uptake of testing and vaccinations, particularly when encouraged by trusted sources. For example, future research may investigate how resources such as free COVID-testing kits, educational flyers, and web toolkits may be tailored for families and promoted by faith-based organizations and religious leaders. Faith-based leaders and organizations have been leveraged for diverse populations to improve a range of health outcomes (e.g., primary prevention, general health maintenance, cardiovascular disease, and cancer) [28].

Several themes and sub-themes identified in this study aligned with levels of the social-ecological model (i.e., individual, interpersonal, institutional, and community) [22]. These barriers and strategies can guide future multi-level interventions and inform responses to future pandemics and crises. Our findings indicated that individual- and interpersonal-level interventions alone, e.g., to increase knowledge and information-seeking behavior, previously emphasized for racial/ethnic minority populations during the pandemic [29], might not be adequate to reduce vaccination and testing uptake disparities. Interventions that address family, community, and institutional barriers are warranted to aid future pandemic responses. For example, keywords such as “No ID required” and community member testimonies may be investigated as part of vaccine and testing messages.

The limitations of this study include that our findings may not be generalizable to other rural Latino populations. Selection bias was inevitable, as we only collected data from those who were interested in and willing to participate. Social desirability bias was another limitation, as the HSC promotora program manager and HSC project director conducted focus groups. As this study was cross-sectional, our findings do not reflect the attitudes of the communities sampled at different stages throughout the COVID-19 pandemic, which has rapidly evolved. Instead, our results reflect attitudes during the early stages of vaccine rollout in Florida, during the peak lockdown periods. Future research must investigate how these attitudes may have evolved, as social-ecological factors, such as vaccine availability and policy, have changed. Moreover, Florida’s rural Latino population is highly diverse, so future research should investigate the role of cultural identity on vaccine uptake [30]. Future research is also needed to better understand the barriers and strategies specific to undocumented populations. Despite these limitations, this study adds to the literature by incorporating diverse community perspectives, including key informants, promotoras, and community members, at the height of the COVID-19 pandemic.

## 5. Conclusions

In conclusion, the social-ecological barriers and strategies identified in this study can add to the information collected from other rural populations and be used to guide future interventions and inform responses to future pandemics and disasters. Although some individual and interpersonal barriers identified in this study (e.g., fear and misinformation) may be similar to the barriers in the general population, many institutional and community barriers (e.g., lack of accessibility and documentation requirements) were specific to rural Latino communities. Many strategies that may be leveraged to overcome such barriers should be unique to rural Latino communities (e.g., community and family resilience). As Latinos experience more socioeconomic hardship, stress, and health risk despite living longer than White and Black individuals [31], the multilevel barriers and strategies identified in this study point to potential targets for intervention to reduce health disparities for this population. Future public health intervention and pandemic response for rural Latino communities should incorporate multiple levels of intervention, including addressing the role of the family, involving trusted community members, and ensuring fair and consistent policies.

## Data Availability

Data generated during this study are available on reasonable request.
